# Effect of the Baltimore Experience Corps Intervention on Memory: Moderation by Neighborhood Characteristics

**DOI:** 10.1093/geroni/igaf122.948

**Published:** 2025-12-31

**Authors:** Kyle Moored, Michael Desjardins, Vijay Varma, Qian-Li Xue, Michelle Carlson

**Affiliations:** Johns Hopkins Bloomberg School of Public Health, Baltimore, Maryland, United States; Johns Hopkins University, Baltimore, Maryland, United States; Clinical and Translational Neuroscience Section, National Institute on Aging, Baltimore, Maryland, United States; Johns Hopkins University, Baltimore, Maryland, United States; Johns Hopkins Bloomberg School of Public Health, Baltimore, Maryland, United States

## Abstract

Neighborhood socioeconomic status (nSES) and walkability may promote cognitive health in later life by providing opportunities for active engagement. Yet, structural disparities have led many older adults to reside in more disadvantaged neighborhoods, especially minoritized groups. Experience Corps was developed to encourage sustained activity for older adults through intergenerational volunteering in local elementary schools. We examined whether Experience Corps improved memory performance and whether this association was moderated by nSES and walkability. Participants were from the Baltimore Experience Corps Trial (n = 659, mean±SD age=67±5.9), which tested the 2-year efficacy of Experience Corps versus an active control. Neighborhood measures included tract-level walkability (e.g., transit access, service/commercial density) and nSES (e.g., poverty, unemployment). Individuals were categorized into combined walkability/nSES groups using median splits of each index: low walkability/high nSES (39%), high walkability/high nSES (12%), high walkability/low nSES (38%), low walkability/low nSES (11%). Memory was assessed using the Rey Auditory Verbal Learning Test (delayed recall) at baseline and 24 months (post-intervention). We fit mixed effects models with individual-level random intercepts and slopes and adjusted for age, sex, education, and health conditions. Experience Corps had no effect on memory performance in the overall sample (p>.05). While the intervention effect on memory did not differ significantly across all neighborhood strata (p-interaction=.12), it was significantly greater for the low walkability/low nSES group versus the low walkability/high nSES group, 1.2 (95% CI: -0.2, 2.6) vs. -0.6 (95% CI: -1.3, 0.2), p=.03. Experience Corps may provide structured opportunities that promote memory especially for aging adults in less resourced neighborhoods.

